# Reflecting the Quality Degradation of Engine Oil by the Thermal Diffusivity: Radiative and Nonradiative Analyses

**DOI:** 10.3390/ma16020773

**Published:** 2023-01-12

**Authors:** Vijayakumar Gokul, Mohanachandran Nair Sindhu Swapna, Dorota Korte, Sankaranarayana Iyer Sankararaman

**Affiliations:** 1 Department of Optoelectronics, University of Kerala, Trivandrum 695581, Kerala, India; 2 Laboratory for Environmental and Life Science, University of Nova Gorica, Vipavska 13, SI-5000 Nova Gorica, Slovenia

**Keywords:** engine oil, thermal diffusivity, thermal lens technique, oil degradation, quality monitoring

## Abstract

Ageing of engine oil is an important issue determining the engine life and performance. The present work attempts to delineate the ageing-induced changes in engine oil through the mode-mismatched dual-beam thermal lens (MMDBTL) technique and other conventional spectroscopic techniques. For the analyses, engine oil samples were collected after every 200 km of runtime. As the thermal diffusivity is related to the nonradiative deexcitation upon optical absorption, comprehensive radiative and nonradiative analyses were carried out. The Ultraviolet-Visible, Fourier transform infrared, and Nuclear magnetic resonance spectroscopic analyses point to the structural modification as a result of the breaking of the long-chain hydrocarbons into ketones, aldehydes, esters, and other compounds. This modifies the absorption pattern, which can also be understood from the nonlinear refractive index study using the Z-scan technique. The compositional variations associated with the degradation upon ageing, the length of the hydrocarbon chain, and the formation of newer molecules account for the enhancement of the thermal diffusivity revealed through the MMBDTL techniques. The complementary nature of the radiative and nonradiative emission is understood from the fluorescence study. Thus, the study reveals the possibility of thermal diffusivity measurement as an effective tool for the quality monitoring of engine oil.

## 1. Introduction

Emissions from automobiles play a significant role in urban air pollution worldwide. The pollutants, such as soot (15%), partially burned hydrocarbons (22.5%), and oxides of nitrogen (14.5%), carbon (32.8%), sulphur (1.4%), and other greenhouse gases (13.8%), in the air emitted from internal combustion engines adversely affect the environmental equilibrium [1,2,3]. Enriching the engine tribology by reducing the friction and wear between the moving parts limits fuel consumption and atmospheric pollution. The engine tribology and heat transfer are mainly controlled by engine oils, which act as a lubricant film between the metal contacts. As a dynamic fluid, engine oil parameters play a vital role in the optimum working condition of an automobile engine [4,5,6]. Engine oil comprises 95–70% of base oil, with different mixtures of hydrocarbons and additives [5,7,8]. Sejkorova et al. [7] pointed out the importance of engine oil in ensuring friction-free motion between the moving surfaces by maintaining an optimum viscosity, thermal stability, oxidation resistance, corrosion retardation, and the removal of residue and contaminants from the engine. According to Tripathi et al. [5], due to ageing, the engine becomes overheated during running, and the oil undergoes thermochemical degradation through high-temperature reactions. As a result of the chemical change due to heating, the residues of burned fuel, condensed water, metal wear particles of the engine, dust, and soot trigger the ageing of the engine oil [7]. Therefore, the appropriate use of engine oil can enhance engine efficiency and durability, which reduces fuel consumption [9]. A literature report revealed that as the rate of oil degradation depends on engine conditions and operation cycles, the engine’s conditions can be indirectly monitored through the quality of the engine oil [10]. 

Techniques employing electromagnetic waves, such as Fourier transform infrared (FTIR), Ultraviolet-Visible (UV-Vis), Photoluminescence (PL), and Nuclear magnetic resonance (NMR) spectroscopy, have emerged as promising tools for quality monitoring in petrochemical and automobile industries, rather than the conventional techniques such as viscosity measurements, the laser flash test, the tribometer test [6], thermogravimetric analysis, and total acid and base number analysis [5,11]. The simplicity of sampling and testing, faster spectral acquisition and manipulation, and ease in tracking samples’ quality degradation increase its competitive advantages over the conventional methods [12,13]. The spectral visualization of the molecular changes in the samples can be effectively monitored using FTIR analysis [14]. Since 1983, IR spectroscopy has been used as a routine tool to understand the quality of lubricant oils [13]. The electronic transitions in materials due to the optical absorption from 200 nm to 800 nm can be analysed using UV-Vis spectroscopy. The UV-Vis spectrum also gives information about the formation and degradation of chromophores in the material. The radiative relaxation from the excited state followed by the optical absorption provides relevant information regarding a material’s energy bands, which can be probed by PL spectroscopy [12]. The optical absorption may also result in optical nonlinearity, which can be easily studied using the Z-scan technique [15,16]. NMR spectroscopy can provide relevant information regarding the chemical environment of the hydrogen and carbon atoms in complex hydrocarbon matrices [17]. Besides these radiative analyses, nonradiative analyses are also widely employed in quality monitoring. 

With the advent of lasers, photothermal spectroscopy has risen as a sensitive technique for material characterizations. Among the various photothermal techniques, thermal lens (TL) spectroscopy has become a standard nondestructive and noncontact method to measure the optical and thermal properties of materials [18,19]. The technique is capable of detecting a temperature variation of 10^−4^ °C to 10^−6^ °C [20]. The method relies on the deexcitation-initiated refractive index change resulting in the formation of a thermal lens, which can be probed by another laser beam [19]. When the pump beam itself is used for probing the TL, the configuration is known as a single-beam TL setup, and when the pump and probe beams have different laser sources, the configuration is called a dual-beam TL setup [18,19]. Among various TL configurations, the mode-mismatched dual-beam (MMDB) setup, which uses a larger probe beam waist than the pump beam at the sample, provides the highest sensitivity and accuracy [18]. The present work delineates the application of laser-induced MMDBTL in the quality monitoring of engine oils through thermal diffusivity measurements. An attempt was also made to correlate the thermal diffusivity of the samples with the results of the radiative analyses.

## 2. Materials and Methods

**Figure 1:**
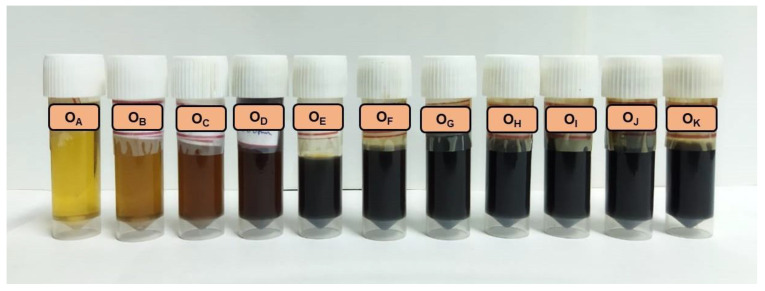
Engine oil samples.

The semi-synthetic four-stroke engine oil SAE 10W-30 used in a 150-cc spark-ignition petrol engine was collected after every 200 km of runtime and was investigated in the present study. The fresh sample was labelled as O_A,_ and the engine oil collected after traversing 200, 400, 600, 800, 1000, 1200, 1400, 1600, 1800, and 2000 km was denoted as O_B_, O_C_, O_D_, O_E_, O_F_, O_G_, O_H_, O_I_, O_J_, and O_K_, respectively. The gradual decrease in the transparency accompanied by the visual colour change due to ageing can be observed in Figure 1.

A detailed analysis of the characteristic changes in the engine oil due to ageing is essential to understand its physiochemical and thermo-optical properties. A PerkinElmer’s FTIR spectrometer was used to study the modes of vibration of the samples in the wavenumber range 4000 cm^−1^ to 400 cm^−1^. The optical absorbance and fluorescence of the samples were analysed using the Jasco V550 UV-Vis absorption spectrometer and Horiba Flurolog TCSPC PL spectrometer, respectively, to understand the ageing-induced variations. A Bruker Avance III HD 400 MHz one bay FT-NMR spectrometer was used to record the NMR spectra of the engine oil samples dissolved in deuterated chloroform. 

**Figure 2:**
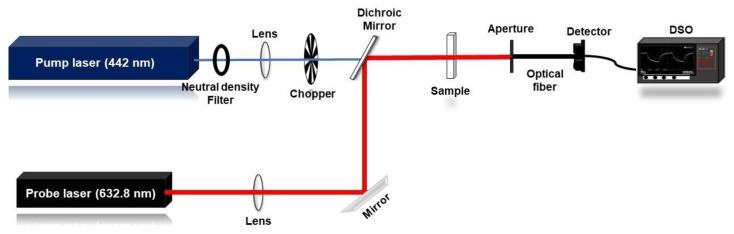
Schematic diagram of the mode-mismatched dual-beam TL setup.

The thermal stability of engine oil relies on the base oil composition and the decomposition of the additives incorporated in it. The thermal behaviour of engine oil varies accordingly due to its usage. The variation in the thermal diffusivity of the oil indirectly points out the physiochemical modification [5], which can be probed using the laser-based TL method. An MMDBTL setup in a collinear configuration, shown in Figure 2, was employed to investigate the thermal diffusivity of the samples.

A Kimmon IK series Helium–Cadmium (He-Cd) gaussian laser (wavelength (λe) 442 nm, energy 80 mW) was used as the pump source. A neutral density filter was used to regulate the power of the pump beam. A low-power Helium–Neon (He-Ne) laser (wavelength (λp) 632.8 nm, energy 2 mW) was used as the probe beam. The samples were taken in a thin glass cuvette of a path length of 3 mm. The beams were focused by separate convex lenses, as shown in Figure 2, and were combined to follow a collinear path through the sample using a dichroic mirror. The He-Cd laser beam was intensity modulated at 1 Hz using an electromechanical chopper SRS-540. A filter was introduced in the emergent beam path to block the pump beam so that only the probe beam reached the detector. $\omega_{e}$ and $\omega_{1p}$ represent the pump and probe radii at the centre of the sample, respectively. The intensity drop at the pump beam centre emerging through the TL was fed to a digital storage oscilloscope (Teledyne DSO, 500 MHz) through a photodetector setup using an optical fibre. The Shen model [21] gives the central beam intensity as Equation (1).

(1) $$I\left(t\right)\;=\;I\left(0\right)\;\left\{\left[1\;-\;\frac{\theta }{2}\;tan^{-1}\;\left(\frac{2mV}{[\left(1+2m{)}^{2}+V^{2}\right]\left(\frac{t_{c}}{2t}\right)+1+2m+V^{2}}\right)\right]{\;}^{2\;}\right\}$$

*I(t)* and *I(0*) indicate the probe beam central intensity at time t and time 0, respectively, θ is the probe beam phase shift, and $t_{c}$ is the characteristic time constant. The parameter θ depends on the absorbed photothermal energy ($P_{th}$), the absorption coefficient (*A*) of the sample, the optical path length (*l*), the temperature-dependent refractive index gradient ($\frac{dn}{dT}$), the thermal conductivity of the sample (*K*), and the probe beam wavelength ($\lambda_{p}$), through Equation (2).

(2) $$\theta \;=\;-\frac{P_{th}\;Al\;\frac{dn}{dT}}{K\lambda_{p}}$$

The ‘m’ in Equation (1) denotes the degree of mode mismatching and can be represented as $m={\left(\frac{\omega_{1p}}{\omega_{e}}\right)}^{2}$. If$\;z_{1}\;$is the separation distance from the probe beam waist (radius $\omega_{0p}$) to the centre of the sample and $z_{c}=\frac{\pi \omega_{0p}{}^{2}}{\lambda_{p}}$, the parameter *V* in Equation (1) is given by $\frac{z_{1}}{z_{c}}$. The parameters θ and $t_{c}$ are obtained by fitting the TL signal using Equation (1), from which the thermal diffusivity (*D*) can be calculated using Equation (3).

(3) $$D=\frac{\omega_{e}{}^{2}}{4t_{c}}$$

**Figure 3:**
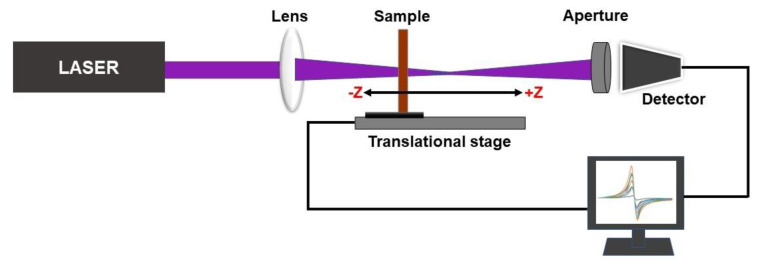
Schematic of the closed aperture Z-scan setup.

Studies on nonlinear optics reveal how intense light interacts with a nonlinear medium. Understanding the nonlinear behaviour, such as the nonlinear refractive index, nonlinear absorption, self-defocusing, and high harmonic generation of material through laser illumination, is essential to categorization for various photonic and optoelectronics applications [22]. A 100 mW variable power diode pumped solid state laser (Stradus, Vortran laser technology) of wavelength (*λ*) 405 nm was focused tightly using a convex lens of focal length (*f*) = 28.6 cm to the sample. Stepper motor-controlled translation of the sample was carried out from −z to +z (−8 cm to +8 cm). The laser beam transmittance through the nonlinear medium as a function of the sample position was measured using a highly sensitive far-field photodetector by keeping an aperture in front of it. The scan started from a distance where the irradiance was low. It then passed through the focal point, where the self-defocusing increased resulting in the broadening of the beam at the aperture, and a reduction in transmittance was observed [15]. The excitation beam waist radius ($\omega_{0})$ at the focal point was obtained as 19.3 µm, using the relation $\omega_{0}=\frac{f\lambda }{d}$, where the laser beam diameter was *d* at the lens surface [16]. The schematic representation of the closed aperture Z scan setup is shown in Figure 3. 

The sample thickness (*L*) was kept below the Rayleigh distance $z_{0}$.

(4) $$z_{0}=\frac{k\omega_{0}{}^{2}}{2}$$

where *k* is the wave vector. The normalized transmittance (*T(z)*) in a closed aperture Z scan is expressed as

(5) $$T\left(z\right)=1-\frac{4x∆\emptyset_{0}}{\left(x^{2}+9\right)\left(x^{2}+1\right)}$$

where x is the ratio between z and z_0_, and $∆\emptyset_{0}$ is the on-axis phase shift, which was obtained by fitting the experimental data with Equation (5). The nonlinear refractive index (*n_2_*) can be calculated using the values of on-axis intensity at the focus (*I*), the effective length of the sample ($L_{eff})=\frac{1-e^{\alpha L}}{\alpha }$, and the linear absorption coefficient (*α*) using the equation

(6) $$n_{2\;}=\frac{∆\emptyset_{0\;}\lambda }{2\pi IL_{eff}}$$

For the Z-scan analysis, individual samples were prepared by mixing 20 µL engine oil (O_A_ to O_K_) in 3 mL of benzene. Benzene was selected as the solvent because it has no absorption at 405 nm. Thus, the nonlinear behaviour of the sample was completely due to the contribution of the engine oil.

## 3. Results and Discussion

**Figure 4:**
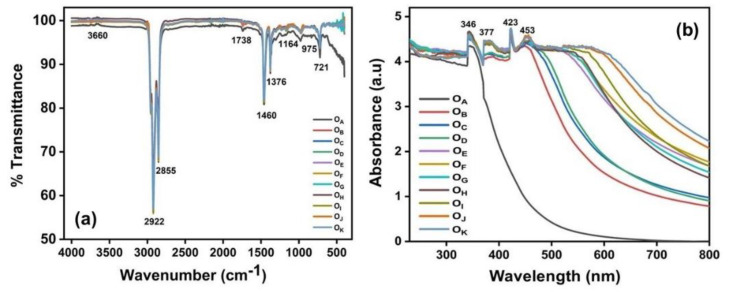
(**a**) FTIR spectra and the (**b**) UV-Vis spectra of the samples.

Engine oil degradation is an intricate process. Variations in the additives, oxidants, contaminants (soot, water, and other residues), physiochemical parameters (viscosity, temperature, and acidity) trigger the quality degradation of the engine oil [5,7]. FTIR spectroscopy is a popular method used to analyse the structural modification of a material. The FTIR spectra of the fresh and aged samples are shown in Figure 4a. 

The IR bands in the region 2922 cm^−1^, 2855 cm^−1^ and 1460 cm^−1^, 1376 cm^−1^, and 721 cm^−1^ were assigned to the mixture of hydrocarbon compounds with a short carbon chain and the alkyl C-H vibrations, respectively [23,24]. The phenolic antioxidant depletion band variations can be observed at 3660 cm^−1^ [25]. The intensity fluctuations in the transmittance value for peaks at 1738 cm^−1^, 1164 cm^−1^, and 975 cm^−1^ pointed out the variations in the viscosity improvers and wear-resistant additives due to ageing [23]. The peak at 1738 cm^−1^ can be attributed to the oxidation byproduct compounds, such as esters, ketones, and carboxylic acids. The peak also represented the stretching vibrations of C=O [26]. The variation in the IR peak intensity at 721 cm^−1^, assigned to the aromatic stretching of the CH bonds, was either due to the reduction in the high molecular weight products or the hydrocarbon polymerization reactions occurring with the ageing of the engine oil [27]. Though a large difference was not visible between the FTIR spectra of the fresh and aged samples, the decrement in the peak intensity corresponding to the phenolic antioxidants indicated the formation of different oxidation products with ageing. 

**Figure 5:**
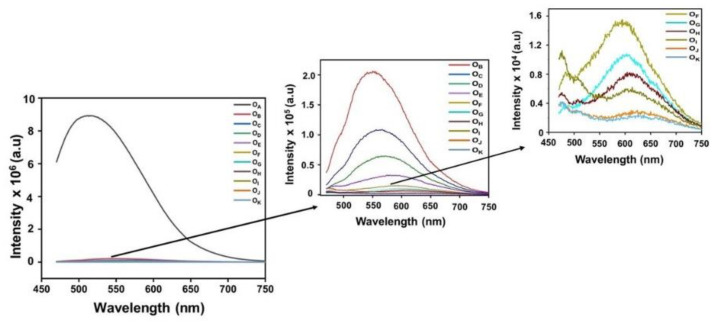
PL spectra of the samples for the excitation wavelength of 442 nm.

How ageing affects the optical absorption behaviour of engine oil is understood from the interaction of the bonding and nonbonding orbitals of material with electromagnetic radiation [28]. The literature [29] reports that the engine oil becomes contaminated by mixing the blowby of combustion gas through worn piston ring gaps. The UV-Vis spectrum of the samples, displayed in Figure 4b, showed a bathochromic shift in the shoulder, indicating the transformation upon ageing. While the sample O_A_ contained only one peak at 346 nm, due to the *n-*π* transitions of the C=O bonds in the oil [30], the ageing resulted in the emergence of additional peaks at 377 nm, 423 nm, and 453 nm. The peaks were due to the degradation of the long-chain hydrocarbons resulting in the formation of chromophoric moieties [24]. The bathochromic shift in the UV-Vis absorption spectrum suggested the formation of newer unsaturated π electron groups, which were responsible for absorption at a higher wavelength. The broadening of the absorption peaks on ageing at each wavelength could be credited to the conjugation of double bonds [31]. If the continued breakage of long-chain hydrocarbons did not occur during ageing, the absorbance level would have increased. However, this did not happen. This confirmed the formation of newer molecular groups, which was responsible for the progressive redshift of the absorption peaks [32,33], as shown in Figure 4b. The absorption rate of electromagnetic radiation by a sample is proportional to the amount of the substances present in the sample. Thus, UV-Vis spectroscopic analysis gives direct information regarding the concentration of the absorption species through the strength of the intensity. From Figure 4b, it is evident that the spectral area increased with ageing, indicating the increase in the decomposition byproducts in the oil. Fluorescence study is a sensitive tool for analysing the photochemical transformations in engine oil. Figure 5 shows the PL spectra of the samples excited at a wavelength of 442 nm, recorded in the range 450–750 nm. 

**Figure 6:**
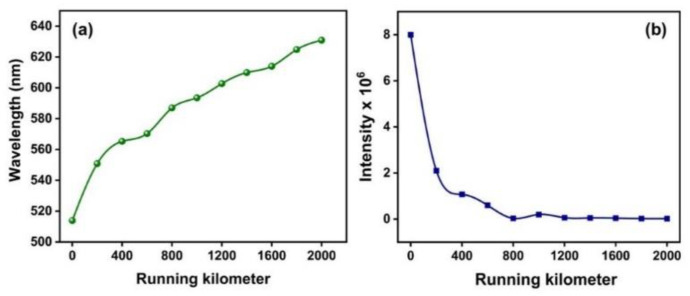
(**a**) Emission peak shift and (**b**) emission peak intensity variation with running kilometres for 442 nm excitation.

From Figure 5, it is evident that the emission decreased with ageing, which could be attributed to the breaking of the long-chain hydrocarbons as evidenced by the UV-Vis absorption spectrum. Figure 6a,b show the redshift in the emission wavelength and intensity reduction in the peak at 513 nm for the 442 nm excitation, respectively.

**Figure 7:**
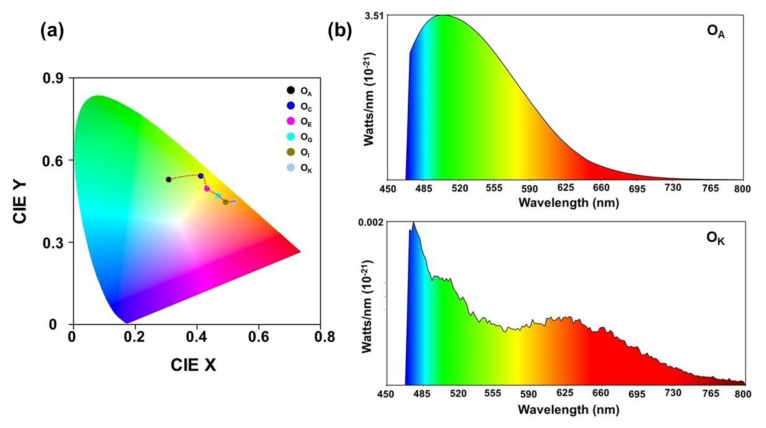
(**a**) The CIE plot of samples—O_A_, O_C_, O_E_, O_G_, O_I_, and O_K_ and the (**b**) power spectrum for the samples—O_A_ and O_K._

The shift towards the higher wavelength was due to the increase in the concentration of the oil and the aggregation of the contaminants, which can be attributed to the collisional energy transfer, the fluorescent quenching, and the inner filter effect [32,33]. The progressive redshift and the intensity reduction in the peak at 513 nm suggested the optical densification as a result of the increase in the contaminant concentration. This was due to the variations in the aromatic polar–polycyclic hydrocarbons degraded to molecular fragments with different masses and the inner filter effect [32]. Thus, the redshift observed in the UV-Vis spectra, as well as the PL spectra, was a direct indication of the degradation of the fresh engine oil. The extent of the shift represented the optical densification of the sample due to ageing. The link between the PL emissions and human colour perception is represented using an International Commission on Illumination (CIE) plot [34,35]. As the CIE representation determines the colour of the fluorescent emission from a sample, it is widely considered as a qualitative tool for assessing the ageing of engine oil [36]. The emissions from the samples for the excitation wavelength of 442 nm are pictorially represented in Figure 7a.

The CIE coordinates for the samples O_A_, O_C_, O_E_, O_G_, O_I,_ and O_K_ were obtained as (0.308, 0.529), (0.412, 0.531), (0.432, 0.495), (0.469, 0.470), (0.493, 0.447), and (0.521, 0.448), respectively, with the emissions moving from the green to the red region. The ageing-induced distribution of the optical energy over the spectrum for the samples O_A_ and O_K_ at 442 nm excitation can be observed from the power spectrum shown in Figure 7b.

**Figure 8:**
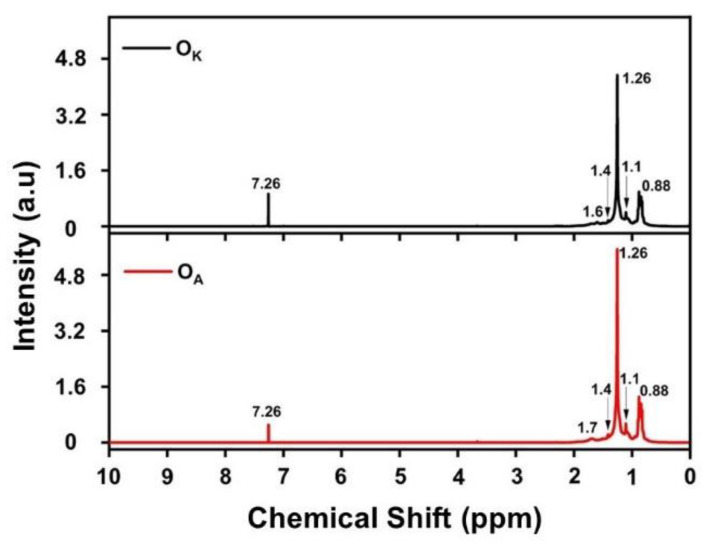
The NMR spectrum of O_A_ and O_K._

The nuclear magnetic resonance method gives an insight into the chemical process that happens during ageing. The NMR is an analytical method through which chemical change can be quantified [37]. The ^1^H NMR spectra of the O_A_ and O_K_ recorded at 400 MHz in the range of 0 to 10 ppm are shown in Figure 8.

**Figure 9:**
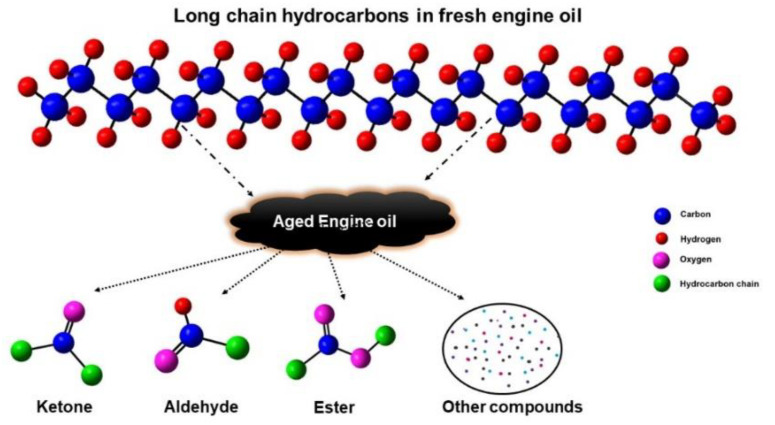
Schematic illustration of the breaking of the long-chain hydrocarbons in aged engine oil.

The spectral peak at 7.26 ppm was associated with functional groups such as esters, heterocyclic aromatics, alcohols, and other compounds corresponding to the base oil degradation [23,26]. The 89% enhancement of the 7.26 ppm peak intensity indicated the formation of newer particles during this process. The peaks ranged from 1.4 ppm to 1.7 ppm due to the overlapping peaks of the CH and CH_2_ groups. The 0.88–1.1 ppm peak was attributed to the presence of terminal methyl groups [8,38]. The predominant signal in the ^1^H spectra represented the protons of the CH_2_ groups in alky chains and the CH_3_ end groups [39]. The NMR analysis confirmed the disintegration of the long-chained hydrocarbons due to ageing. Thus, the observation supported well the other spectroscopic studies carried out for the confirmation of the ageing-initiated degradation of the engine oil. The schematic representation (Figure 9) shows the breaking of the long-chain hydrocarbons into ketones, aldehydes, esters, and other compounds.

**Figure 10:**
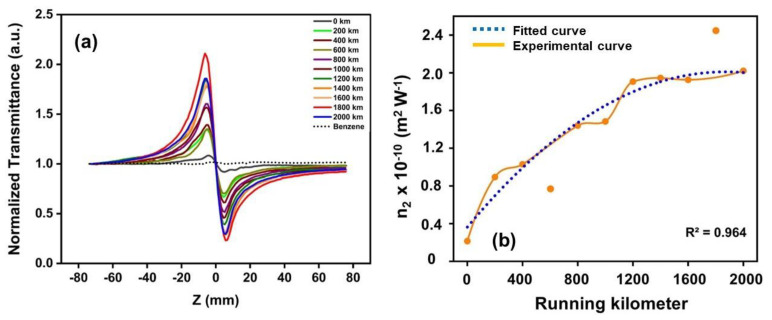
(**a**) The normalized transmittance versus z plot of the samples and the (**b**) nonlinear refractive index variation of the samples with respect to ageing.

The changes in the engine oil due to ageing (O_A_ to O_K_) change the optical absorption and nonlinear refractive index n_2_, which can be analysed by the closed aperture Z-scan technique. The variation in the transmitted intensity was analysed across the focal plane. The normalized transmittance versus z plot of the samples O_A_ to O_K_ for the laser power of 10 mW is shown in Figure 10a. 

The prefocal transmittance maxima followed by a postfocal transmittance minimum in Figure 10a indicated the negative nonlinear refractive index of the samples. This agreed well with the literature [15]. The n_2_ of the samples was obtained by curve fitting Equation (6), and its variation with respect to ageing, shown in Figure 10b, shows a second-order polynomial relation (R2 = 0.964) with the running kilometres. The increasing trend of the n_2_ with ageing due to the optical densification via the consequent degradation of the long-chained hydrocarbons and the contamination of the engine oil resulted in the increase in residues, such as soot, disintegrated hydrocarbons, ketones, aldehydes, and carboxylic acids, as observed in the FTIR, UV-Vis, PL, and NMR spectroscopic analyses. 

**Figure 11:**
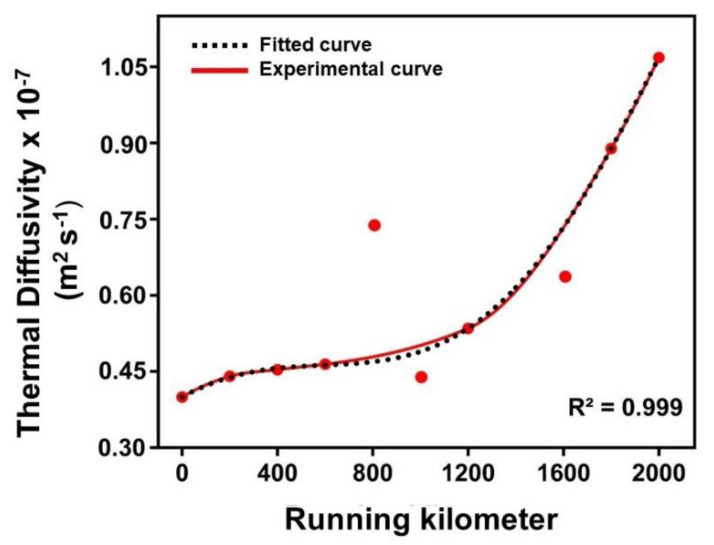
The thermal diffusivity variation in the engine oil with running kilometres.

To understand the ageing of engine oil due to the regular operation of a petrol engine, the MMDBTL technique that finds a wide range of applications in trace detection was adopted in this work. As the presence of contaminants in engine oil can alter its thermo-optical and physiochemical properties, it is essential to monitor these changes to preserve and improve engine performance. The thermal diffusivity variation in the engine oil, with respect to the running kilometres of the engine, measured using the MMDBTL setup, is shown in Figure 11.

The thermal diffusivity increased with the increase in running kilometres. The presence of residues significantly influenced the thermal behaviour of the engine oil, similar to the nanoparticles in nanofluids [6,40,41]. The increase in the volumetric fraction of the ageing-induced contaminants led to the aggregation of the nanoparticles in the engine oil, which increased the effective path length of the propagation of heat energy through it. This was the reason for the enhancement in the thermal diffusivity, as given in the Sankar–Swapna model of the generalized theory of thermal conductivity [42]. As the intensity of the emission peak of the PL spectra, shown in Figure 6b, decreased, the nonradiative output increased, which in turn accounted for the increase in the thermal diffusivity and thereby suggests the thermal diffusivity as a measure of the quality of the engine oil.

## 4. Conclusions

The work analysed the ageing-induced changes in the semi-synthetic four-stroke engine oil SAE 10W-30 used in a 150-cc spark-ignition petrol engine through the MMDBTL technique and other conventional spectroscopic techniques. The variation in the thermal diffusivity, a thermophysical parameter, was targeted to monitor the quality degradation of the engine oils. As thermal diffusivity is related to nonradiative relaxation upon optical absorption, a comprehensive study on the radiative and nonradiative analyses was carried out. The FTIR, UV-Vis, and NMR spectroscopic analyses revealed the structural modification as a result of the breaking of the long-chain hydrocarbons into ketones, aldehydes, esters, and other compounds, which in turn accounted for the nonlinear refractive index studied using the Z-scan technique. The bathochromic shift in the UV-Vis absorption spectrum asserted the formation of newer unsaturated π electron groups, which were responsible for the absorption at a higher wavelength. The complementary nature of the radiative and nonradiative emission was revealed through the photoluminescence study. The study suggests that the ageing-induced compositional variations are responsible for the thermal diffusivity enhancement and thereby reveal the possibility of thermal diffusivity measurement as an effective tool for the quality monitoring of engine oil.

## Data Availability

The data that support the finding of this study are available from the corresponding author upon reasonable request.

## Abbreviations

FTIR	Fourier transform infrared
UV-Vis	Ultraviolet-Visible
PL	Photoluminescence
NMR	Nuclear magnetic resonance
TL	Thermal lens
MMDB	Mode-mismatched dual-beam
SAE	Society of Automotive Engineers
DSO	Digital storage oscilloscope
He-Cd	Helium–Cadmium
He-Ne	Helium–Neon
CIE	International Commission on Illumination
**List of symbols**	
°C	Degree Celsius
h	Hour
cc	Cubic capacity
km	Kilometre
cm	Centimetre
mm	Millimetre
nm	Nanometre
mW	Milliwatt
λ_e_	Pump beam wavelength (nm)
λ_p_	Probe beam wavelength (nm)
Hz	Hertz (s^−1^)
$\omega_{e}$	Pump beam radius at the sample (µm)
$\omega_{1p}$	Probe beam radius at the sample(µm)
$\omega_{0p}$	Probe beam radius at beam waist (µm)
$\omega_{0}$	Excitation beam waist radius (µm)
I_0_	Intensity of probe beam at time = 0
I_(t)_	Intensity of probe beam at time = t
θ	Probe beam phase shift
t_c_	Characteristic time constant
K	Thermal conductivity (W·m^−1^ K^−1^)
D	Thermal diffusivity (m^2^ s^−1^)
n	Refractive index
ΔT	Change in temperature
$\frac{\mathrm{dn}}{\mathrm{dT}}$	Refractive index gradient
P_th_	Absorbed photothermal energy (J)
A	Absorption coefficient of the sample (m^−1^)
l	Path length of the cuvette (mm)
m	Degree of mode mismatching
f	Focal length of the convex lens (cm)
d	Laser beam diameter (mm)
$∆\emptyset_{0}$	On-axis phase shift
n_2_	Nonlinear refractive index (m^2^·W^−1^)
$L_{\mathrm{eff}}$	Effective length of the sample
α	Linear absorption coefficient (cm^−1^)
µL	Microlitre
